# Structured Background Modeling for Hyperspectral Anomaly Detection

**DOI:** 10.3390/s18093137

**Published:** 2018-09-17

**Authors:** Fei Li, Lei Zhang, Xiuwei Zhang, Yanjia Chen, Dongmei Jiang, Genping Zhao, Yanning Zhang

**Affiliations:** 1 Shaanxi Key Lab of Speech & Image Information Processing (SAIIP), School of Computer Science and Engineering, Northwestern Polytechnical University, Xi’an 710129, China; feixiang145@mail.nwpu.edu.cn (F.L.); zhanglei211@mail.nwpu.edu.cn (L.Z.); chenyanjia@mail.nwpu.edu.cn (Y.C.); jiangdm@nwpu.edu.cn (D.J.); ynzhang@nwpu.edu.cn (Y.Z.); 2 School of Computers, Guangdong University and Technology, Guangzhou 510006, China; zhaoyinpin888@163.com

**Keywords:** background modeling, block-diagonal structure, spatial-spectral dictionary learning, anomaly detection, hyperspectral imagery

## Abstract

Background modeling has been proven to be a promising method of hyperspectral anomaly detection. However, due to the cluttered imaging scene, modeling the background of an hyperspectral image (HSI) is often challenging. To mitigate this problem, we propose a novel structured background modeling-based hyperspectral anomaly detection method, which clearly improves the detection accuracy through exploiting the block-diagonal structure of the background. Specifically, to conveniently model the multi-mode characteristics of background, we divide the full-band patches in an HSI into different background clusters according to their spatial-spectral features. A spatial-spectral background dictionary is then learned for each cluster with a principal component analysis (PCA) learning scheme. When being represented onto those dictionaries, the background often exhibits a block-diagonal structure, while the anomalous target shows a sparse structure. In light of such an observation, we develop a low-rank representation based anomaly detection framework that can appropriately separate the sparse anomaly from the block-diagonal background. To optimize this framework effectively, we adopt the standard alternating direction method of multipliers (ADMM) algorithm. With extensive experiments on both synthetic and real-world datasets, the proposed method achieves an obvious improvement in detection accuracy, compared with several state-of-the-art hyperspectral anomaly detection methods.

## 1. Introduction

A hyperspectral image (HSI) shows a powerful ability to distinguish different materials, because of collecting abundant spectral characteristics of materials within hundreds or even thousands of bands covering a wide range of wavelengths [1]. Different from traditional images, each pixel in an HSI contains a spectral vector where each element represents the reflectance radiance in a specific band [2]. Thus, HSIs can be employed to facilitate a variety of applications such as target detection and classification [3,4,5,6,7,8]. In hyperspectral target detection, the key is to obtain the inherent spectral information or characteristics of targets. However, some inevitable influence often hinders the accurate acquisition of target spectral information, such as the absorption and scattering of the atmosphere, subtle effects of illumination, and the spectral response of the sensor [9].

To sidestep this problem, plenty of methods turn to investigate anomaly detection, which aim at distinguishing plausible targets from the background, without introducing any supervised information of the targets [10,11,12]. In general, hyperspectral anomaly detection can be regarded as an unsupervised binary classification problem between the background class and the anomaly class [13]. Background modeling methods can be roughly classified into two categories, namely traditional background statistics modeling-based methods and recent background representation-based methods.

In the background statistics modeling-based methods, it is assumed that the background comes from a mathematical distribution. For example, the Reed-Xiaoli (RX) algorithm [14] assumes the background to follow a multivariate normal distribution. With the mean vector and covariance matrix estimated from some selected sample pixels, the Mahalanobis distance between the test pixel and the mean vector is employed as a detector, which considers pixels with distances larger than a threshold as an anomaly. RX methods have two typical versions: the global RX, which estimates the background statistics from all pixels in the image, and the local RX, which infers the background distribution with pixels lying in local windows. Although this RX detector is mathematically simple and of low computational complexity [15], they suffer from three aspects of limitations. Firstly, an HSI often contains various materials, which makes the background cluttered and difficult to be well depicted by a single multivariate norm distribution. Secondly, the noise corruption in the HSI often causes inaccurate estimation of the background statistics [16]. Thirdly, inversing the estimated high-dimensional covariance matrix in RX is often ill-conditioned and unstable, especially with a small amount of sample pixels [17,18]. To overcome these limitations, some improved methods have been proposed. For example, Catterall et al. [19] developed a Gaussian-mixture-based anomaly detector to model the multi-mode background. Carlotto [20] divided the cluttered background into homogeneous clusters and then assumes each cluster follows a Gaussian distribution. Other improved cluster-based or Gaussian-mixture-based anomaly detection approaches have also been developed [21,22]. To represent the background beyond spectral space, kernel RX (KRX) was proposed to estimate the background statistics in a high-dimensional kernel feature space with a radial basis function (RBF) Gaussian kernel function [23]. Inspired by this kernel theory, Jin Zhou et al. developed a novel cluster kernel RX algorithm for anomaly detection [24]. Its key idea is to group background pixels into clusters and then apply a fast eigendecomposition algorithm to generate the anomaly detection index. Although the multi-mode characteristics of background have been described based on the cluster strategy, it is still a method based on statistical modeling, which cannot accurately describe the background. Another nonlinear local method is based on the support vector data description (SVDD) [25]. The SVDD operator estimates an enclosing hypersphere around the background in a high-dimensional feature space and treats pixels that lie outside the hypersphere as outliers. Although these aforementioned background statistics modeling-based methods are theoretically simple, the difficulty in accurately modeling the cluttered background limits their capacity in anomaly detection.

Recently, witnessing the success of representation method in a wide range of applications [26,27], some methods commence at investigating the background representation-based anomaly detection method. Different from background statistics modeling methods, these methods aim at distinguishing the anomaly pixels from the background in a representation space. By doing this, they are able to sidestep the difficulty in modeling the complicated distribution of background and show promising performance in anomaly detection. For example, Chen et al. [3] introduce the sparse representation theory into hyperspectral target detection for the first time. They assume that the background and anomaly pixels can be well represented by the corresponding background and anomaly dictionaries, respectively. Reconstruction error is then employed to locate the targets. However, in the anomaly detection, there is no supervised information of target, nor is an anomaly dictionary established. To address this problem, Wei and Qian [10] propose a collaborative-representation-based detector. It is inspired by the observation that each pixel in the background can be approximately represented by its spatial neighborhoods, whereas anomalies cannot. Li et al. [28] propose an anomaly detection method by using a background joint sparse representation (BJSR) model, which estimates the adaptive orthogonal background complementary subspace to adaptively select the most representative background bases for the local region. Zhu and Wen [29] employ the endmember extraction model to construction background over-complete dictionary, the anomaly targets are then extracted by the residual matrix based on sparse representation model. Ma et al. [30] propose a multiple-dictionary sparse representation anomaly detector based on the cluster strategy. However, these representation methods do not consider the global correlation among pixels in an HSI [7,16].

To overcome this issue, a low-rank representation [31] scheme has been proposed for hyperspectral anomaly detection. In this scheme, it is assumed that background pixels are low-rank while anomalies are sparsely distributed in the image scene. The anomalies can then be separated from the background by solving a constrained regression problem. Based on this basic concept, many studies have been investigated. For example, Sun et al. [32] utilized a Go Decomposition (GoDec) algorithm [33] to decompose an HSI into a low-rank background matrix with a sparse matrix. Anomalies are then detected within the sparse matrix based on the Euclidean distance. Zhang et al. [15] proposed a low-rank and sparse matrix decomposition-based mahalanobis distance method for hyperspectral anomaly detection. It extracts anomaly targets in the background part by using the mahalanobis distance after decomposing the original HSI. Qu et al. [34] and Wang et al. [35] proposed a low rank representation method based on the spectral unminxing strategy. However, the structure information was ignored when performing low rank decomposition on the unmixing result. Xu et al. [16] and Niu et al. [36] also discussed the hyperspectral anomaly detection problem based on the low-rank representation theory. Although the low-rank representation scheme can improve the detection performance, it still suffers from certain limitations. Firstly, the low rank constraint fails to explicitly model the multi-mode structure of the background [37,38,39,40]. In addition, due to spectral variation, the background is often full rank.

To address these problems, we propose a novel block-diagonal structure-based low-rank representation framework for hyperspectral anomaly detection. Through representing the spatial– spectral characteristics of each pixel with a local full-band patch, we divide all pixels into several homogeneous clusters to conveniently exploit the multi-mode structure of the background. Then, a PCA learning method is adopted to learn a spatial-spectral dictionary for each cluster. Since these PCA dictionaries extract the major structure information of each cluster as well as suppress the information (e.g., anomalies), background pixels are prone to be well represented by the dictionary learned from the cluster they belong to, while the anomalies cannot be well represented by most dictionaries. Therefore, when being represented by those learned dictionaries, the background often exhibits an obvious block-diagonal structure, while the anomaly shows a sparse structure. Inspired by the fact that matrices of a block-diagonal structure often imply a low-rank property [41], a low-rank representation model is built to represent the background on those dictionaries. Through solving this model by a standard alternating direction method of multipliers (ADMM) algorithm, an HSI is decomposed into a low-rank background part with a sparse anomaly one. Since anomalies often occur as a few pixels embedded in the local homogeneous backgrounds [42], they have the sparse property. Thus, the anomaly target can be determined by calculating the $\ell_{2}$ norm of the residual vector of each pixel in the obtained sparse matrix. Compared with the existing low-rank representation-based methods, the main contributions of this work are threefold:

1. **Multi-Mode Background Representation.** Most previous low-rank representation-based methods model the background as a whole and assume the background exhibits low-rank characteristic [43]. Although this concept provides effective prior information for the background, it fails to explicitly model the multi-mode characteristics of background; e.g., due to containing various materials, the background often exhibits different clusters of intra-cluster similarity as well as inter-cluster dissimilarity. To explicitly capture the multi-mode characteristics of the background, we divide the input HSI into several homogeneous clusters through classifying pixels with spatial–spectral features. A PCA dictionary is then learned for each cluster. When being representing with those dictionaries, the multi-mode characteristics of the background are cast into a specific block-diagonal structure, which can be explicitly modeled.
2. **Block-Diagonal Structure Modeling.** A block-diagonal structure is often exploited in subspace clustering [44] or classification [45] to depict the affinity of samples from various classes. To our knowledge, this is the first attempt to employ a block-diagonal structure in modeling the background of an HSI for anomaly detection. Introducing a block-diagonal structure brings twofold advantages. Firstly, it can explicitly depict the multi-mode characteristics of the background in the representation space. Secondly, the block-diagonal structure is more robust than the low-rank structure. A low-rank structure depends on the feature consistency of pixels, and a slight variation may cause the background to be full-rank, while a block-diagonal structure depends on the feature dissimilarity of pixels, which is more robust to feature variation.
3. **Spatial–Spectral Feature based Dictionary Learning.** In this study, we exploit the block-diagonal structure of the background in a clustering-based representation space that is determined by the dictionaries learned on all clusters. Thus, it is crucial to construct a data-driven and robust dictionary. To this end, we first represent each pixel by the spatial-spectral feature in a local full-band patch for robust clustering. A PCA learning scheme is then adopted to learn the dictionary for each cluster, which guarantees that the major structure of each cluster is captured and the block-diagonal structure of the background is revealed.

The rest of this paper is organized as follows. In Section 2, we give a detailed description of the proposed Block-Diagonal Structure Based Low-Rank Representation (BDSLRR) model and the patch-based dictionary construction method. Section 3 is the optimization procedure for the proposed model. Both simulated and real-world data experimental results and analyses are provided in Section 4, and Section 5 concludes the paper.

## 2. the Block-Diagonal Structure-Based Low-Rank Representation For Anomaly Detection

In this section, we first introduce the block-diagonal property for anomaly detection, and the proposed model is explained in detail. After introducing the spatial-spectral feature-based dictionary learning method, we show the entire flow of the proposed algorithm.

### 2.1. Block-Diagonal Property for Anomaly Detection

The aim for anomaly detection is to distinguish interesting targets from the local or global background without any prior spectral information of the targets. Since the anomalous pixels are unknown and few, a reasonable way is to accurately model the background. However, due to the cluttered imaging scene, an HSI often contains different categories of materials which exhibits various spectra, and the corresponding background is not homogeneous but multi-mode. Therefore, for accurate anomaly detection, it is crucial to exploit the multi-mode structure in the background. A promising way to capture the multi-mode structure is to apply the clustering method, which specializes in collecting similar pixels into a homogeneous cluster, while dispersing different pixels into various clusters, thus representing the multi-mode structure with the resulted different clusters. Some studies [20,46,47] have employed clustering methods to analyze the multi-mode statistical distributions of the background in anomaly detection.

In this study, we propose to incorporate the clustering method and dictionary learning scheme to depict the multi-mode structure of the background in the representation-based detection framework. Through clustering the background, we obtain several homogeneous clusters, and different clusters exhibit an obvious discrepancy. Thus, given an appropriate dictionary learned from a specific cluster, only pixels belonging to this cluster can be well represented, while the anomalous pixels cannot be well represented by most dictionaries. Thus, when being represented on the concatenation of all dictionaries learned from each cluster, the representation matrix of the background exhibits an obvious block-diagonal structure.

To clarify this point, we first decompose the input HSI X into a background part as well as anomaly part, which can be formulated as

(1) $$\begin{array}{c} X=X_{bg}+E, \end{array}$$

where $X_{bg}$ is the background part and E is the anomaly part. As discussed above, the background can be represented by a reasonable dictionary while the anomaly E cannot. Thus, we can represent $X_{bg}=\mathrm{DZ}$ and reformulated Equation (1) as

(2) $$\begin{array}{c} X=\mathrm{DZ}+E \end{array}$$

where $D=[D_{1},D_{2},\cdots ,D_{k}]$ contains *k* background sub-dictionaries which is learned from each cluster independently, $D_{i}$ corresponds to *i*-th sub-dictionaries, and Z is the background representation matrix.

To exploit the block-diagonal structure in Z, we first permute columns in X as $X^{*}=\left[X_{1}^{*},X_{2}^{*},\cdots ,X_{k}^{*}\right]$, $X_{i}^{*}$ represents the *i*-th cluster, and each column in $X_{i}^{*}$ denotes the spectra of a specific pixel. The background part $X_{bg}$ can then be reformulated as $X_{bg}^{*}={\mathrm{DZ}}^{*}$, where $Z^{*}$ is the corresponding permuted representation matrix. Let $\left\{S_{1},S_{2},\cdots ,S_{k}\right\}$ be a collection of *k* subspaces, each of which has a rank (dimension) of $r_{i}>0$. We then give the following theoretical results [41].

**Theorem** **1.** Without loss of generality, given $X_{bg}^{*}={\mathrm{DZ}}^{*}$, assume that $D_{i}$ is a collection of $m_{i}$ samples of the i-th subspace $S_{i}$, $X_{i}$ is a collection of $n_{i}$ samples from $S_{i}$, and the sampling of each $D_{i}$ is sufficient such that rank($D_{i}=r_{i}$). If the subspaces are independent, then $Z^{*}$ is block-diagonal [41]:

(3) $$\begin{array}{c} Z^{*}=\left[\begin{array}{cccc} Z_{1}^{*} & 0 & 0 & 0\\ 0 & Z_{2}^{*} & 0\; & 0\\ 0 & 0 & \ddots & 0\\ 0 & 0 & 0\; & Z_{k}^{*} \end{array}\right] \end{array}$$

where $Z_{i}^{*}$ is an $m_{i}\times n_{i}$ coefficient matrix with $\mathrm{rank}\left(Z_{i}^{*}\right)=\mathrm{rank}\left(X_{i}\right),\forall i$.

In this study, each cluster stands for one subspace and these subspaces (*clusters*) are independent due to the spectral diversities. Therefore, according to Theorem 1, with clustering and permuting the original HSI, the background representation matrix $Z^{*}$ will exhibit a block-diagonal structure.

### 2.2. Block-Diagonal Structure Based Low-Rank Representation Model

As discussed in the previous subsection, we utilized the cluster method to exhibit the multi-mode structure of the background and be able to more accurately represent the background. Each cluster we obtained is sharing common features (e.g., spectral) and its elements show strong similarities. Intuitively, each pixel should be represented by the base elements of its corresponding cluster, then the ideal representation of data will have a block-diagonal structure as follows:

(4) $$\begin{array}{c} Z=\left[\begin{array}{cccc} Z_{1} & 0 & 0 & 0\\ 0 & Z_{2} & 0\; & 0\\ 0 & 0 & \ddots & 0\\ 0 & 0 & 0\; & Z_{k} \end{array}\right] \end{array}$$

where $Z_{i}$ is the representation matrix of the *i*-th background cluster corresponding to the dictionary or feature of the *i*-th backgroud cluster, and *k* is the cluster number. According to the property of the block-diagonal matrix [48], the following theorem is obtained.

**Theorem** **2.** *The rank of a block diagonal matrix equals the sum of the ranks of the matrices that are the main diagonal blocks.*

With the above theorem, the rank of Z in Equation (4) can be calculated by the following:

(5) $$\begin{array}{c} \begin{array}{c} rank\left(Z\right)=rank\left(Z_{1}\right)+rank\left(Z_{2}\right)+\cdots +rank\left(Z_{k}\right). \end{array} \end{array}$$

Since the *i*-th cluster has a strong inner similarities, the *i*-th representation matrix $Z_{i}$ also has this characteristic. $Z_{i}$ then exhibits a low-rank property. Based on this rule, all the $Z_{i}\left(i=1,\cdots ,k\right)$ have a low-rank property. The representation matrix Z thus obviously exhibits a low-rank property according to Equation (5), which is consistent with the characteristics of the HSI background.

Based on the above discussion, the proposed Block-Diagonal Structure Based Low-Rank Representation (BDSLRR) is implemented by integrating the multi-mode structure background and sparse anomaly targets as follows:

(6) $$\begin{array}{c} \min_{Z,E}\;\mathrm{rank}\left(Z\right)+\lambda {∥E∥}_{2,1},\\ s.t\;X=DZ+E \end{array}$$

where rank(·) denotes the rank function, the parameter λ>0 is used to balance the effects of the two parts, and ${∥\cdot ∥}_{2,1}$ is the $\ell_{2,1}$ norm defined as the sum of the $\ell_{2}$ norm of the column of a matrix. $X=\left[x_{11},\cdots ,x_{1n_{1}},\cdots ,x_{k1},\cdots ,x_{kn_{k}}\right]\in R^{b\times n}$ is a sorted 2-D HSI matrix according to the cluster processing (supposing that there are *k* clusters for the HSI, $x_{ij}\left(i=1,\cdots ,k;j=1,\cdots ,n\right)$ is the *j*-th pixel of the *i*-th cluster, $n_{1}+\cdots +n_{k}=n$ is the total number of samples, *b* is the number of hyperspectral bands), DZ denotes the background part, D is the background dictionary learned by each cluster, Z denotes the background block-diagonal representation coefficients, and E denotes the remaining part corresponding to the anomalies [16]. The reason for the sparsity of anomalies in Equation (6) is that the dictionary D stands for background characteristics only and cannot be utilized to represent anomaly targets reasonably. Moreover, there are very low amounts of anomaly targets in the data X compared with the background pixels, thus the anomaly targets may have sparsity property rather than a low-rank property. Consequently, it is reasonable to add the sparse constraint into anomaly targets as shown in Equation (6).

After getting the sparsity matrix E, the role of the *i*-th pixel can be determined as follows:

(7) $$\begin{array}{c} r\left(x_{i}\right)={∥{\left[E\right]}_{:,i}∥}_{2}=\sqrt{\sum_{j}{\left({\left[E\right]}_{j,i}\right)}^{2}}{\;}_{<}^{>}\;\delta \end{array}$$

where ${∥{\left[E\right]}_{:,i}∥}_{2}$ denotes the $\ell_{2}$ norm of the *i*-th column of E, δ is the segmentation threshold, and, if $r(x_{i})>\delta$, $x_{i}$ is determined as the anomaly pixel; otherwise, $x_{i}$ is labeled as the background.

It should be noted that there are some essential advantages in our model compared with the former low-rank-representation-based methods [15,16,32,36]. Firstly, we adopt the cluster method to describe the background, which can exploit background information and characteristics more accurately. This kind of detailed feature has not been considered in the former low-rank-based methods, which regarded the background as a whole. Secondly, the block-diagonal structure is utilized to represent the multi-mode structure information of the background based on the cluster result. The structure information is helpful for improving detection performance, which is ignored in the former low-rank-based methods. Thirdly, we employ local spatial–spectral constraints to construct the background dictionary, which can extract background features efficiently. The next section will provide a detailed explanation. In brief, our model can capture better features of the HSI in both global and local aspects than the former low-rank based methods. Moreover, the later experimental results will also prove that the proposed model can achieve better detection performance.

### 2.3. Spatial–Spectral Feature-Based Dictionary Learning

Generally, the background dictionary has a great impact on the representation-based anomaly detection methods [3,16,26,49,50,51]. Based on previous studies, the background dictionary often needs to meet three main conditions to be considered well-constructed. Firstly, the obtained dictionary should represent all background clusters, so as to represent the diversity of the background. Secondly, the dictionary must be robust to the anomaly targets and noise, meaning that the final dictionary should maintain the background feature while excluding other contaminations. Lastly, it can display the spatial-spectral feature, which is a specific and important feature for the HSI. In previous work [3,49], these conditions have not been integrated together, so the learned dictionary cannot reasonably represent the background.

**Figure 1:**
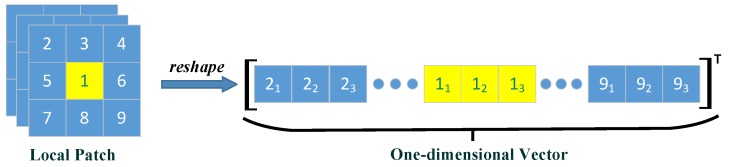
Schematic illustration of the patch-based spatial–spectral constrain. Pixel 1 is the center, and pixel 2∼9 are the neighborhood pixels. The bottom-right number of each pixel is its corresponding spectral band number. As there are 3 bands and the patch size is 3×3, the size of the reshaped one-dimensional vector is (3×3×3)×1=27×1. According to this rule, supposing that the original HSI cube is rows×columns×nbands and that the patch is n×n, then the 2-D patch-based matrix is [nbands×n×n]×[rows×columns].

To overcome this issue, we adopt a spatial–spectral feature-based dictionary learning method. Although anomaly detection techniques rely upon the spectral difference between pixels, there are spatial correlations for neighbourhood pixels, so collaboration considering the spatial–spectral feature can effectively improve the detection result [40,52,53,54]. Inspired by this, we replaced each single pixel with its surrounding pixels to form a local patch before the clustering and dictionary learning step. Specifically, for a given hyperspectral pixel *x*, we selected an n×n size 3-D local patch whose center is *x*. This 3-D patch was then reshaped to be a one-dimensional vector. Figure 1 takes a 3×3 size patch and 3 spectral bands to show a brief example of this approach. The new reshaped matrix gives a good integrated representation of spatial-spectral features simultaneously.

We then utilized the *k*-means [55] method to divide the patch-based data into k clusters, and each cluster could represent one background material roughly. In this way, the multi-mode characteristics of the background could be well exhibited by selecting a reasonable k (k should be larger than the true number of ground material clusters in order to make sure that the k cluster represents all ground materials [16]).

When clustering the background, the anomaly targets will be assigned to one of the clusters. To obtain a clean background dictionary, it is essential to remove those plausible anomalies during dictionary learning. To this end, we adopted the PCA technique for dictionary learning. It has been shown that the significant components in PCA deliver the major information of the data. In a given cluster, the major information comes from the background pixels. Thus, we removed the less significant components after PCA to eliminate the negative effect of anomalies on the learned dictionary. Finally, we obtained the background dictionary D after using the PCA leaning algorithm for each cluster.

The advantages of spatial-spectral feature-based dictionary learning technique are as follows:

1. A patch-based spatial-spectral feature construction method provides a more robust way of describing the correlations of spatial and spectral within an HSI. The one-dimensional vector shown in Figure 1 contains both spectral information and spatial neighbourhood characteristics, so as to enhance the detection performance of the proposed method.
2. By using the cluster way to represent background, both the diversity and multi-mode structure information of the background can be well described explicitly. Moreover, the low-rank property of the background is enhanced, which is helpful to increase the separability of anomaly targets and the background while solving the BDSLRR model.
3. The PCA learning scheme allows a clean background dictionary to be learned. Although anomalies may be categorized into one background cluster at the beginning, their information can be effectively eliminated from the learned dictionary by neglecting those less significant principal components during PCA learning.

### 2.4. Entire Flow of the Proposed Algorithm

**Figure 2:**
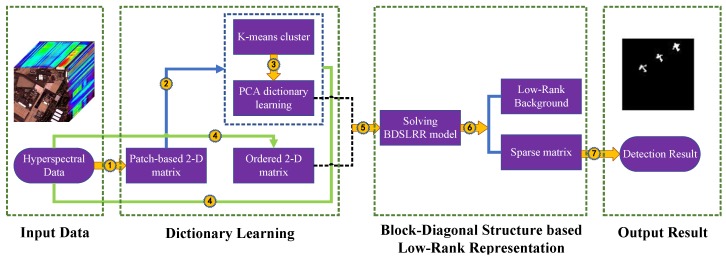
Framework of the proposed method.

According to the introduction above, the entire flow of the proposed method can be shown in Figure 2. It can be seen clearly that the proposed method mainly contains two modules: dictionary learning and block-diagonal structure-based low-rank representation. Given an HSI, we utilized the patch-based strategy, the clustering method, and the PCA method to obtain a spatial-spectral intra-cluster background dictionary. The block-diagonal structure-based low-rank representation model could then be built. By solving this model, we can obtain the sparse matrix containing the targets. As a result, the targets are extracted from this sparse matrix. The detailed steps of the proposed method have been summarized in Algorithm 1.
**Algorithm 1:** The Proposed BDSLRR Method **Input**: Original 3-D Hyperspectral image $X_{original}$.*Step 1*:Reshaping the input 3-D data into patch-based2-D matrix;*Step 2*:Dividing the patch-based 2-D matrix intodifferent clusters;*Step 3*:Getting the dictionary of each cluster by using the PCAlearning method, and obtain the wholebackground dictionary D*Step 4*:According to the dictionary order of each cluster,reshaping and re-ordering the 3-D $X_{original}$ into2-D matrix X so as to correspond with D;*Step 5*:Input X and D into Equation (6), and constructthe BDSLRR model based on multi-modeblock-diagonal structure;*Step 6*:Solve the BDSLRR model, and obtain the low-rankbackground matrix and the sparse matrix E;*Step 7*:Extract anomaly targets from the sparse matrix Eby Equation (7) **Output**: Detection Result.


## 3. Optimization Procedure

This section presents the detailed procedure of how to solve the BDSLRR model. The model in Equation (6) is non-convex and NP-hard. An effective way to mitigate this problem is to relax Equation (6) into the following convex problem:

(8) $$\begin{array}{c} \min_{Z,E}\;{∥Z∥}_{*}+\lambda {∥E∥}_{2,1},\\ s.t\;X=DZ+E \end{array}$$

where the nuclear norm ${∥\cdot ∥}_{*}$ is utilized to replace the original rank regularization. It has been shown that the solution of Equation (6) is equal to that of Equation (8) when some mild conditions hold [41].

In our study, we employed the standard alternative direction method of multipliers (ADMM) to solve the problem in Equation (8). Specifically, we first reformulate Equation (8) as follows

(9) $$\begin{array}{c} \min_{Z,E,J}\;{∥J∥}_{*}+\lambda {∥E∥}_{2,1},\\ s.t\;X=DZ+E,Z=J. \end{array}$$

We can then obtain the following Lagrangian function:

(10) $$\begin{array}{cc} L= & {∥J∥}_{*}+\lambda {∥E∥}_{2,1}+\mathrm{tr}\left(Y_{1}^{T}\left(X-DZ-E\right)\right)\\ & +\mathrm{tr}\left(Y_{2}^{T}\left(Z-J\right)\right)+\frac{\mu }{2}{(∥X-DZ-E∥}_{F}^{2}\\ & +{∥Z-J∥}_{F}^{2}) \end{array}$$

where $Y_{1}$ and $Y_{2}$ are Lagrange multipliers, and μ>0 is the penalty coefficient. Like [41,56], given the Lagrangian function, the detailed steps for solving Equation (10) can be summarized in Algorithm 2. For the detailed derivation for each step, interested readers can refer to [41,56]. Note that the convex problems in Steps 1 and 3 have closed-form solutions. Step 1 is solved via the Singular Value Thresholding (SVT) operator [57], while Step 3 is solved via the following lemma.

**Lemma** **3.** *Let Q be a given matrix. If the optimal solution to*

(11) $$\begin{array}{c} \min_{W}\;\alpha {\left|\left|W\right|\right|}_{2,1}+\frac{1}{2}{\left|\left|W-Q\right|\right|}_{F}^{2} \end{array}$$

is $W^{*}$, then the $t^{th}$ column of $W^{*}$ is

(12) $$\begin{array}{c} {\left[W^{*}\right]}_{:,i}=\left\{\begin{array}{cc} \frac{{\left|\left|{\left[Q\right]}_{:,i}\right|\right|}_{2}-\alpha }{{\left|\left|{\left[Q\right]}_{:,i}\right|\right|}_{2}}Q_{:,i}, & \mathrm{if}\;{\left|\left|{\left[Q\right]}_{:,i}\right|\right|}_{2}>\alpha ;\\ 0, & \mathrm{otherwise}. \end{array}\right.. \end{array}$$

Since there are three blocks (including Z, J, and E) in Algorithm 2, and the objective function of Equation (9) is not smooth, it is difficult to generally ensure the convergence of ADMM [58]. Fortunately, there are actually some guarantees for ensuring the convergence of Algorithm 2. According to the theoretical results in [59], two conditions are sufficient (but may not be necessary) for Algorithm 2 to converge. The first is that the dictionary matrix D is of full column rank. In the proposed method, dictionary D consists of principal components from each cluster. Considering that all clusters are different from each other, principal components from different cluster will be uncorrupted with high probability. Thus, columns in D are uncorrelated, viz., D is of full column rank. The other condition is that the optimality gap produced in each iteration step is monotonically decreasing, viz., the error

(13) $$\begin{array}{c} \epsilon_{k}={\left|\left|\left(Z_{k},J_{k}\right)-\arg\;\min_{Z,J}\;L\right|\right|}_{F}^{2} \end{array}$$

is monotonically decreasing, where $Z_{k}$ (respectively, $J_{k}$) denotes the solution produced at the *k*th iteration, and $\arg\min_{Z,J}\;L$ indicates the “ideal” solution obtained by minimizing the Lagrangian function *L* with respect to both Z and J simultaneously [59]. Based on [59], the convexity of the Lagrangian function can guarantee its validity to some extent, although it is not easy to strictly prove it. As illustrated in [59], ADMM is known to generally perform well in reality. Moreover, our experiments in Section 4 show that ADMM can achieve a good result for our BDSLRR model.

## 4. Experiments and Discussion

This section will verify the feasibility and effectiveness of our proposed method by comparing with 5 state-of-the-art anomaly detection algorithms on four datasets. Additionally, we analyze the sensitivity of relevant parameters and the effectiveness of the spatial–spectral feature-based dictionary learning method.

### 4.1. Comparison Methods

There are 10 state-of-the-art anomaly detection algorithms are employed to evaluate our proposed method.

- The global RX (GRX) [14] algorithm. This method is one of the most typical Gaussian-based anomaly detection algorithm and frequently used to be the benchmark comparison method. We employ an open published MATLAB code [60] for the method in this study.
- The collaborative representation-based anomaly detection algorithm (CRD) [10]. This algorithm is directly based on the concept that each pixel in the background can be approximately represented by its spatial neighborhoods, while anomalies cannot. To estimate the background, each pixel is approximately represented via a linear combination of surrounding samples within a sliding dual window. The weight vector of combination, based on the distance-weighted Tikhonov regularization, has a closed-form solution under the $\ell_{2}$-norm minimization. The anomalies are calculated from the residual image which is obtained by subtracting the predicted background from the original hyperspectral data. Its MATLAB code can been downloaded easily [61].
- The cluster-based anomaly detector (CBAD) [20]. This approach tends to divide data into appropriate clusters. Inside each cluster, a Gaussian mixture model (GMM) is supposed. The Mahalanobis distance is then calculated between the pixel under test and the center of each cluster. Pixels that exceed the threshold are considered anomalies.
**Algorithm 2:** ADMM for BDSLRR 
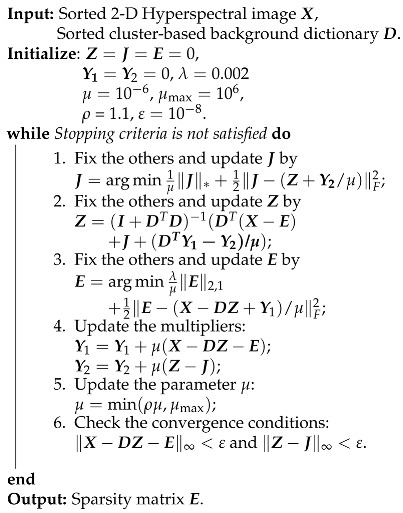

- The background joint sparse representation method for hyperspectral anomaly detection (BJSRD) [28]. This is a newly developed anomaly detection method based on sparse representation. Based on the sparse representation theory, the algorithm utilizes the redundant background information in the hyperspectral scene and automatically deals with the complicated multiple background clusters, without estimating the statistical information of the background.
- The local summation anomaly detector (LSAD) [40] algorithm. This local summation strategy integrates both the spectral and spatial information together. The edge expansion and subspace feature projection operation are included in this strategy to enhance detection performance.
- The low-rank and sparse representation (LRASR)-based anomaly detection method [16]. This is the first time that the low-rank representation (LRR) has been adopted for anomaly detection purposes in an HSI. The background information is characterized by the low rankness of the representation coefficients, and the anomaly information is contained in the residual. When constructing the dictionary, it utilizes the *k*-means method, but it does not consider the spatial– spectral correlation.
- The low-rank and sparse matrix decomposition-based Mahalanobis distance method [15]. This method adopts the GoDec algorithm [62] proposed by Zhou and Tao [33] to solve the low-rank background component and the sparse component. A Mahalanobis-distance-based anomaly detector is used to extract anomalies based on the low-rank background part.
- The orthogonal subspace projection (OSP) anomaly detector [63]. The OSP carries out background suppression, via orthogonal projection, in order to remove the main background structures.
- The local RX anomaly detector [64]. This approach employs a dual window strategy when calculating the Mahalanobis distance for each testing pixel. The inner window is slightly larger than the pixel size, the outer window is even larger than the inner one, and only samples in the outer region are adopted to estimate the covariance matrix.
- The local kernel RX (LKRX) anomaly detector [23]. This approach is similar to the conventional local RX, but every term in the expression is in a high-dimensional kernel feature space with a radial basis function (RBF) Gaussian kernel function, which can be readily calculated in terms of the input data in its original data space.

### 4.2. Dataset Description

In this study, five hyperspectral datasets collected from different instruments are used to evaluate the effectiveness of the proposed detector, and the targets have different sizes and spatial distributions. All these real-world datasets adopted in this study are commonly used in anomaly detection [16,28,40,65,66,67]. We strictly follow the experimental protocol provided by these previous studies.

#### 4.2.1. The Synthetic Hyperspectral Dataset

**Figure 3:**
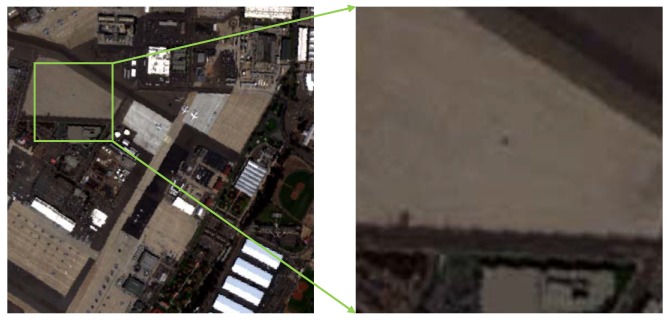
Hyperspectral Data Sets. Left is the original San Diego airport image, and right is the selected area for forming the simulated data.

The synthetic hyperspectral image comes from a real HSI data which was collected by the Airborne Visible/Infrared Imaging Spectrometer (AVIRIS) from the San Diego airport area, CA, USA. It has 224 spectral channels in wavelengths ranging from 370 to 2510 nm, and the spatial resolution is 3.5 m per pixel. A total of 189 available bands of the data were retained in our experiments after removing the bands that correspond to the water absorption regions and that have a low SNR and bad bands (1∼6, 33∼35, 97, 107∼113, 153∼166, and 221∼224). The original size of the dataset is 400×400 pixels. In our study, a region with a size of 100×100 pixels is chosen to form the simulated image. The original image and selected area are shown in Figure 3.

The anomalous pixels are simulated by the target implantation method [68]. Based on the linear mixing model, a synthetic sub-pixel anomaly target with spectral *x* and a specified abundance fraction α is generated by fractionally implanting a desired anomaly with spectral t in a given pixel of background with spectral b as follows:

(14) $$\begin{array}{c} x=\alpha \cdot t+(1-\alpha )\cdot b. \end{array}$$

**Figure 4:**
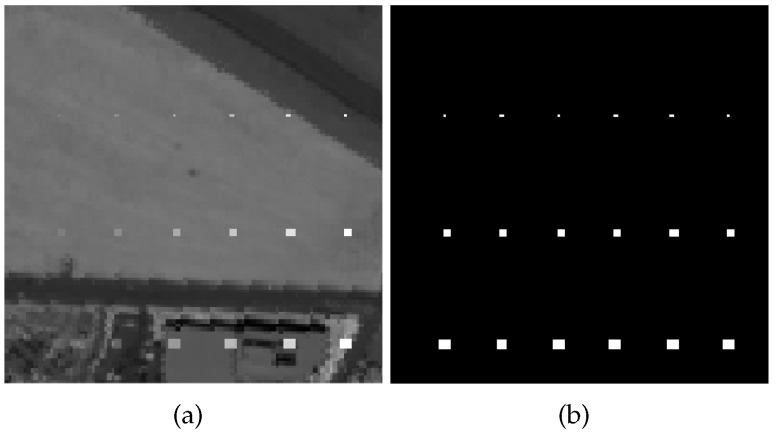
Synthetic Hyperspectral Dataset. (**a**) Synthetic dataset; (**b**) Ground truth of the synthetic dataset.

In the synthetic image, 18 anomalous targets have been implanted. They are distributed in three rows and six columns and the sizes of anomalous are 1×1, 2×2, and 3×3, from top to bottom in each column. In each row, the abundance fractions α are 0.1, 0.2, 0.4, 0.6, 0.8, and 1.0, from left to right. The spectrum of the plane is assumed to be the anomalous spectrum t, which is chosen from the middle left of the whole scene. The simulated data and ground truth are shown in Figure 4.

#### 4.2.2. Real Hyperspectral Datasets

**Figure 5:**
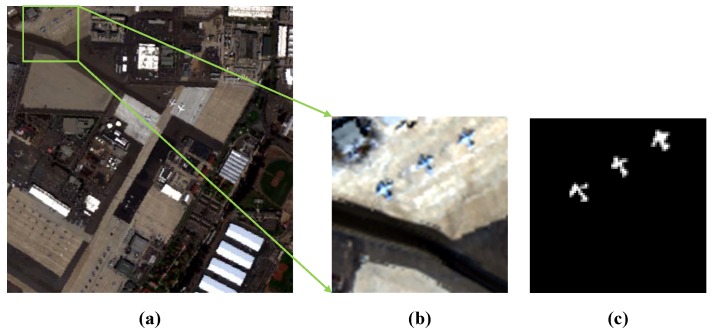
First Real-World Dataset. (**a**) Whole San Diego airport image dataset; (**b**) Upper left of the San Diego airport hyperspectral image; (**c**) Ground truth for the anomaly targets.

The first real-world dataset is also selected from the San Diego Airport image. It is a region with a size of 80×80 pixels chosen from the upper left of the scene as test image. We regard the small aircraft as anomaly targets in this sub-image. The dataset is shown in Figure 5.

**Figure 6:**
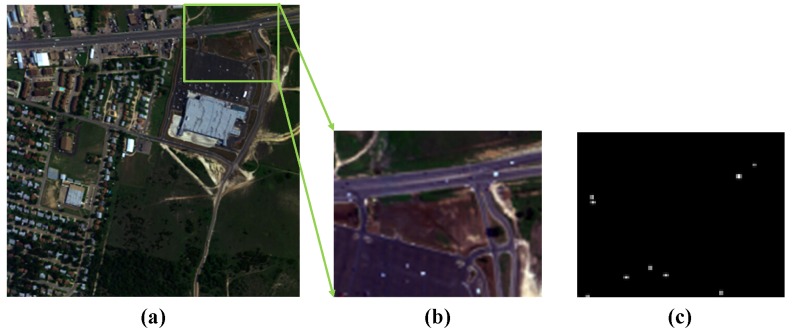
HYDICE hyperspectral image named Urban. (**a**) Whole HYDICE hyperspectral image; (**b**) Upper left of the scene. (**c**) Ground truth for the anomaly targets.

The second real-world dataset is the HYDICE hyperspectral image named Urban [69]. The data has a spectral resolution of 10 nm, a spectral range of 400∼2500 nm, and a spatial resolution of 2×2 m^2^, and there are 307×307 pixels in the entire set of data, with 210 spectral bands, as shown in Figure 6a.

In this study, we only use a sub-image of the data for our experiments. Specifically, we cut out a size of 80×100 sub-image from the upper right of the scene as shown in Figure 6b, and maintain only 160 bands after eliminating the low-SNR and water vapor absorption bands (1∼4,76,87,101∼112,136∼153, and 197∼210). The scene is cluttered with a parking lot and a roadway with 10 man-made vehicles which can be considered as anomaly targets [40] in this image and the reference is shown in Figure 6c.

**Figure 7:**
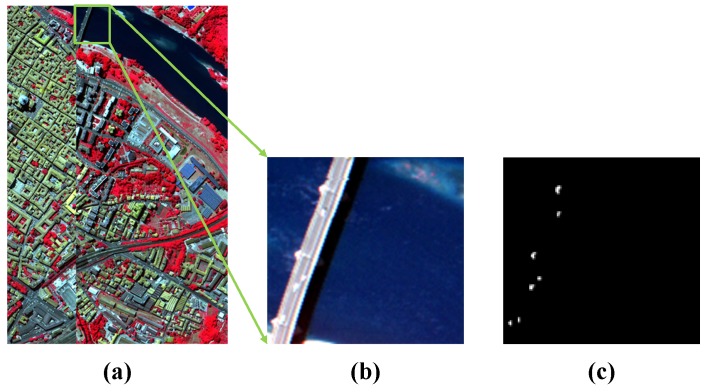
Pavia Center (PaviaC) dataset. (**a**) Whole Pavia Center (PaviaC) dataset; (**b**) Smaller subset of the scene; (**c**) Ground truth for the anomaly targets.

The third real-world dataset is the Pavia Center (PaviaC) dataset was downloaded from the Computational Intelligence Group of the Basque Country University as is shown in Figure 7a [70]. The dataset was acquired by the reflective optics system imaging spectrometer (ROSIS) sensor and has been widely used in many applications [4,71]. The dataset covers the Pavia Center in Northern Italy and has accurate ground truth information. The number of bands in the initial dataset is 115 with 1.3 m spatial resolution covering the spectral range from 430∼860 nm. In the experiment, a smaller subset is segmented from the initial larger image. The subset contains 115×120 pixels and 102 bands after removing low signal-to-noise ratio (SNR) bands. In the false-color image of Figure 7b, three ground objects constitute the background: bridge, water, and shadows. Anomaly pixels representing vehicles on the bridge and the bare soil near the bridge pier also appear in the image scene [72]. The ground truth of the anomalies is shown in Figure 7c.

**Figure 8:**
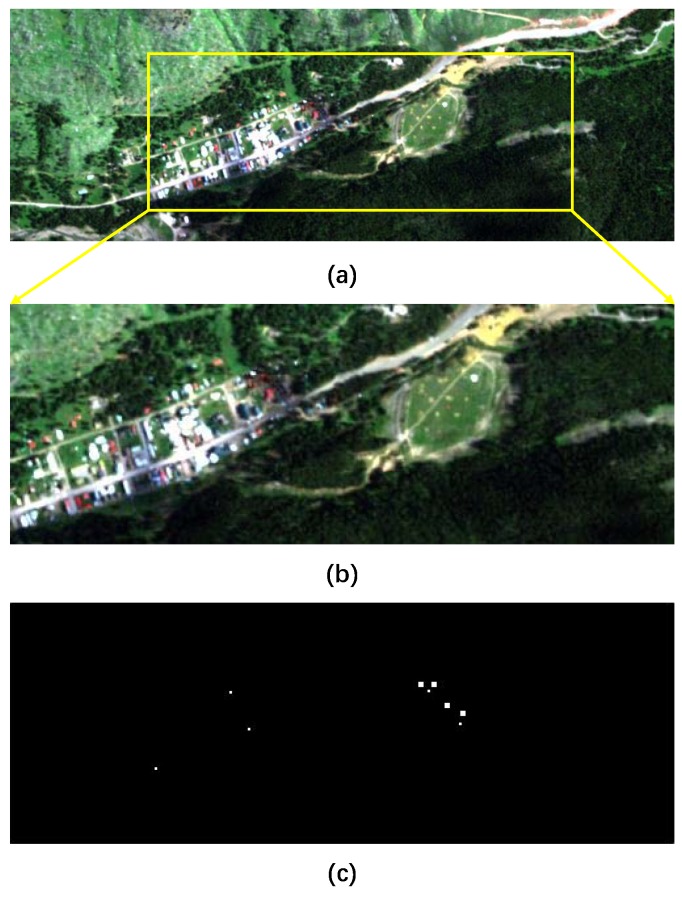
Hyperspectral dataset provided by the *Target Detection Blind Test* project. (**a**) Whole dataset; (**b**) Smaller subset of the scene; (**c**) Ground truth for the anomaly targets.

The forth real-world dataset is provided by the *Target Detection Blind Test* project [73]. This dataset was collected by a HyMap instrument over Cook City in Montana, on July 2006. It (illustrated in Figure 8a has 280×800 pixels in size and 126 spectral bands. The spatial resolution of the data is quite fine, with a pixel size of approximately 3 m. Seven types of targets, including four fabric panel targets and three vehicle targets, were deployed in the region of interest. In our experiment, we crop a subimage of size 183×506, including all of these targets (anomalies) as depicted in Figure 8b. The ground truth of the anomalies is shown in Figure 8c.

### 4.3. Detection Performance

In this section, all detection results (including seven comparison methods and the proposed BDSLRR method) will be presented. To evaluate the detection performance, ROC curves and AUC values are employed. ROC curves can plot the relationship between the false alarm ratio and the detection ratio by taking all possible thresholds based on the target references. The AUC value is calculated with the whole area under the ROC curve, and can be helpful to identify general trends in detector performance [74]. Ideally, a good detection algorithm should distinguish between anomalies and background separating both classes as much as possible. To do that, rare pixels must be marked with notably high scores, while background pixels have almost zero values. However, ROC curves and AUC metrics only indicate that the anomalous pixels have higher values than the background pixels, but do not indicate how “separated” these values are. For this reason, two extra quality metrics will be utilized in this paper: the squared error ratio (SER) and the area error ratio (AER). The lower the SER, the better the algorithm performs. The higher the AER is, the better the algorithm performs. More detailed explanation for these two metrics can be found in [75]. For a fair comparison, each detection map is linearly normalized by its maximum value in the performance evaluation step, and all parameters of each method are optimal. The detailed information of parameters will be shown in the next section.

**Figure 9:**
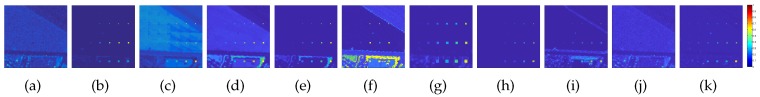
Two-dimensional plots of the detection results obtained by different methods for the synthetic dataset. (**a**) GRX; (**b**) BJSRD; (**c**) LSAD; (**d**) LRASR; (**e**) LSMAD; (**f**) CBAD; (**g**) CRD; (**h**) LRX; (**i**) LKRX; (**j**) OSP; and (**k**) BDSLRR.

**Figure 10:**
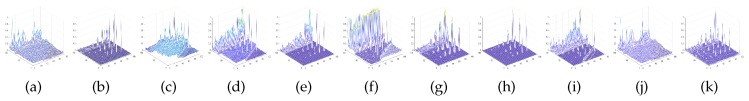
Three-dimensional plots of detection results for the synthetic dataset. (**a**) GRX; (**b**) BJSRD; (**c**) LSAD; (**d**) LRASR; (**e**) LSMAD; (**f**) CBAD; (**g**) CRD; (**h**) LRX; (**i**) LKRX; (**j**) OSP; and (**k**) BDSLRR.

**Figure 11:**
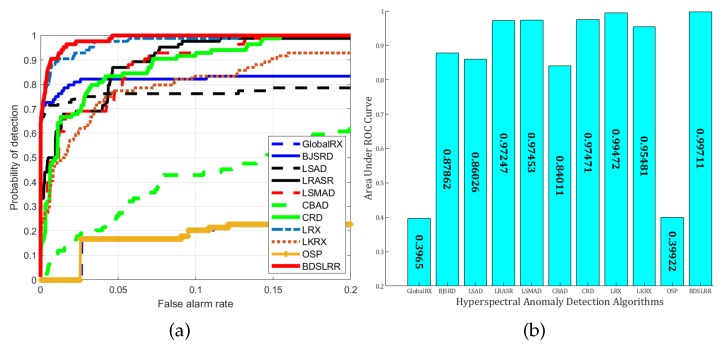
Detection accuracy evaluation for the synthetic dataset. (**a**) ROC curves; (**b**) AUC values.

For the synthetic dataset experiment, the two-dimensional plots of detection results and the three-dimensional plots of detection images of the compared anomaly detection methods and the proposed methods are illustrated in Figure 9 and Figure 10. From these figures, the proposed BDSLRR gives color maps where the anomalies are obvious. The ROC curves of all methods are shown in Figure 11a for illustrative purposes. The AUC scores are provided in Figure 11b. This data contains a high amount of sub-pixel targets. When the traditional GRX method was utilized, these sub-pixel targets were also included in the calculation of the covariance matrix of the background, so the obtained background covariance matrix could not describe the background information accurately. As a result, the detection performance decreased. The AUC value of the proposed BDSLRR is 0.99711, which is larger than GRX, BJSRD, LSAD, LRASR, LSMAD, CBAD, LRX, LKRX, OSP, and CRD algorithms.

**Figure 12:**
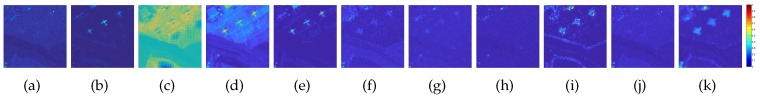
Two-dimensional plots of the detection results obtained by different methods for the San Diego dataset. (**a**) GRX; (**b**) BJSRD; (**c**) LSAD; (**d**) LRASR; (**e**) LSMAD; (**f**) CBAD; (**g**) CRD; (**h**) LRX; (**i**) LKRX; (**j**) OSP; and (**k**) BDSLRR.

**Figure 13:**
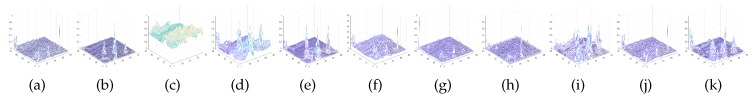
Three-dimensionalplots of the detection results for the San Diego dataset. (**a**) GRX; (**b**) BJSRD; (**c**) LSAD; (**d**) LRASR; (**e**) LSMAD; (**f**) CBAD; (**g**) CRD; (**h**) LRX; (**i**) LKRX; (**j**) OSP; and (**k**) BDSLRR.

**Figure 14:**
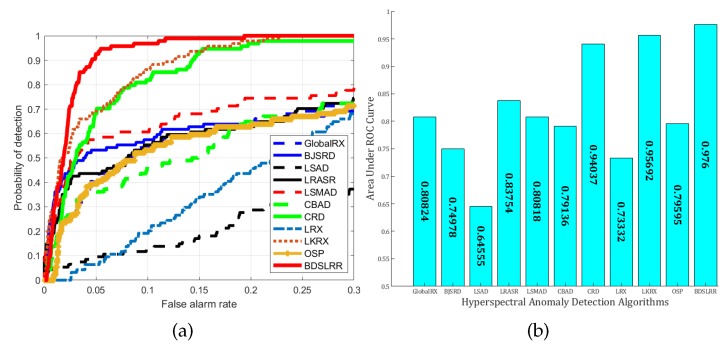
Detection accuracy evaluation for the San Diego dataset. (**a**) ROC curves; (**b**) AUC values.

For the real San Diego dataset, the two-dimensional plots for the detection results and the three-dimensional plots of detection results are shown in Figure 12 and Figure 13. The ROC curves of all the methods are shown in Figure 14a for illustrative purposes. The proposed BDSLRR achieves the highest detection probability for all false alarm rate values. The AUC scores are provided in Figure 14b. The proposed BDSLRR achieves the highest score, and this confirms that the proposed method can outperform the traditional and state-of-the-art detectors.

**Figure 15:**
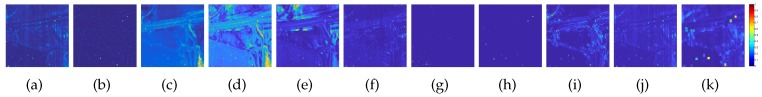
Two-dimensional plots of the detection results obtained by different methods for the Urban dataset. (**a**) GRX; (**b**) BJSRD; (**c**) LSAD; (**d**) LRASR; (**e**) LSMAD; (**f**) CBAD; (**g**) CRD; (**h**) LRX; (**i**) LKRX; (**j**) OSP; and (**k**) BDSLRR.

**Figure 16:**
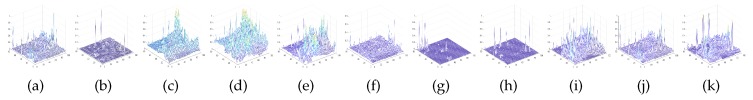
Three-dimensional plots of detection results for the Urban dataset. (**a**) GRX; (**b**) BJSRD; (**c**) LSAD; (**d**) LRASR; (**e**) LSMAD; (**f**) CBAD; (**g**) CRD; (**h**) LRX; (**i**) LKRX; (**j**) OSP; and (**k**) BDSLRR.

**Figure 17:**
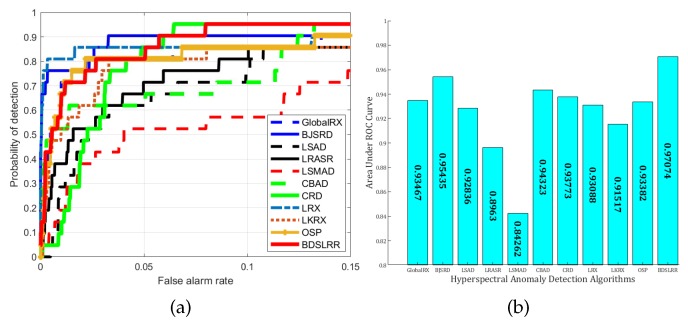
Detection accuracy evaluation for the Urban dataset. (**a**) ROC curves; (**b**) AUC values.

For the real Urban dataset, the detection results and the three-dimensional plots of detection results are shown in Figure 15 and Figure 16. From these figures, it can be seen that the proposed BDSLRR gives a map where the anomalies are obvious. The ROC curves of all the methods are shown in Figure 17a. The AUC scores are shown in Figure 17b. Although the BJSRD gains a higher probability of detection when the false alarm rate ranges from 0 to 0.05, the proposed BDSLRR achieves the highest score among all detectors.

**Figure 18:**
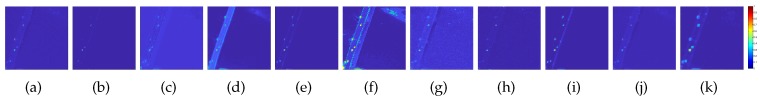
Two-dimensional plots of the detection results obtained by different methods for the Pavia dataset. (**a**) GRX; (**b**) BJSRD; (**c**) LSAD; (**d**) LRASR; (**e**) LSMAD; (**f**) CBAD; (**g**) CRD; (**h**) LRX; (**i**) LKRX; (**j**) OSP; and (**k**) BDSLRR.

**Figure 19:**
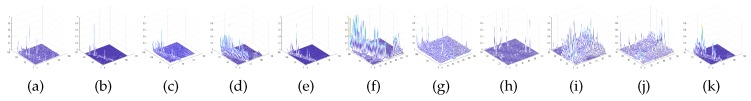
Three-dimensional plots of detection results for the Pavia dataset. (**a**) GRX; (**b**) BJSRD; (**c**) LSAD; (**d**) LRASR; (**e**) LSMAD; (**f**) CBAD; (**g**) CRD; (**h**) LRX; (**i**) LKRX; (**j**) OSP; and (**k**) BDSLRR.

**Figure 20:**
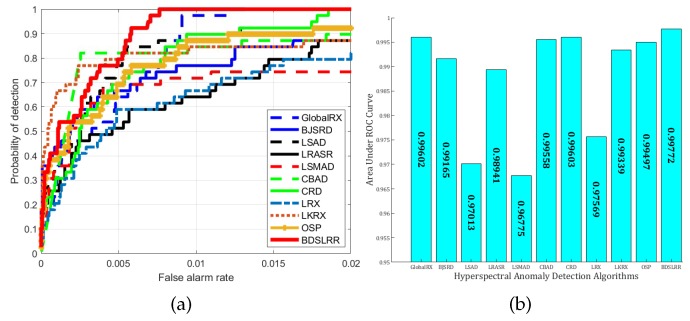
Detection accuracy evaluation for the Pavia dataset. (**a**) ROC curves; (**b**) AUC values.

For the real Pavia dataset, the two-dimensional plots of the obtained detection results are illustrated in Figure 18a–k. The three-dimensional plots of detection results are shown in Figure 19a–k. Compared with other datasets, this data has a less complicated background, so the anomalies are obvious in these figures. All these methods can suppress the background reasonably, but the targets are also suppressed for GRX, LRX, LKRX, OSP, BJSRD, LSAD, and LSMAD methods, and for LRASR, CBAD, and CRD algorithms, the bridge has brought great interference to the target, which decreases the detection performance. The ROC curves and AUC scores of all the methods are shown in Figure 20. Although AUC scores for all the comparison methods are more than 0.95, the proposed BDSLRR achieves the highest values. This verifies the robustness and stability of the proposed method.

**Figure 21:**
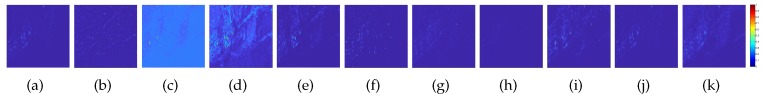
Two-dimensional plots of the detection results obtained by different methods for the *Target Detection Blind Test* dataset. (**a**) GRX; (**b**) BJSRD; (**c**) LSAD; (**d**) LRASR; (**e**) LSMAD; (**f**) CBAD; (**g**) CRD; (**h**) LRX; (**i**) LKRX; (**j**) OSP; and (**k**) BDSLRR.

**Figure 22:**
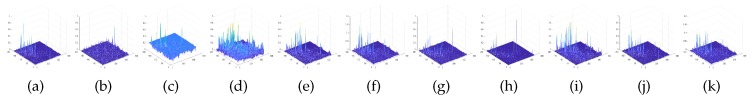
Three-dimensional plots of detection results for the *Target Detection Blind Test* dataset. (**a**) GRX; (**b**) BJSRD; (**c**) LSAD; (**d**) LRASR; (**e**) LSMAD; (**f**) CBAD; (**g**) CRD; (**h**) LRX; (**i**) LKRX; (**j**) OSP; and (**k**) BDSLRR.

**Figure 23:**
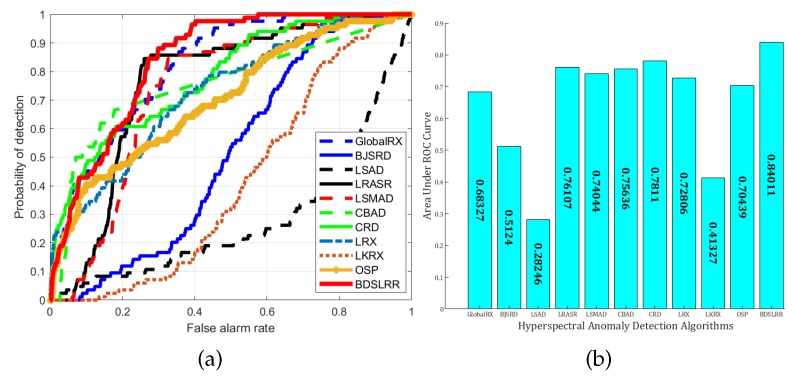
Detection accuracy evaluation for the *Target Detection Blind Test* dataset; (**a**) ROC curves. (**b**) AUC values.

For the real *Blind Test* dataset, the two-dimensional plots for the obtained detection results are illustrated in Figure 21a–k. The three-dimensional plots are shown in Figure 22a–k. The two- dimensional plots demonstrate that GRX, LRX, LKRX, OSP, LSAD, and LSMAD and the proposed method can suppress the background more reasonably than the other methods. However, GRX, LRX, LKRX, OSP, LSAD, and LSMAD fail to consider the multi-mode property and global block structure of the background. Their AUC values are lower than those of the proposed method, shown as Figure 23b. The CBAD exploits the multi-mode property through the clustering strategy as the proposed method does, but it still employs an RX-based detector that cannot exploit the appropriate structure of the background, so its AUC value of 0.75636 is lower than the AUC value of the proposed method, 0.84011. Therefore, these results demonstrate that the proposed method can achieve superior detection performance with the more complicated and larger-sized datasets than the other methods.

**Figure 24:**
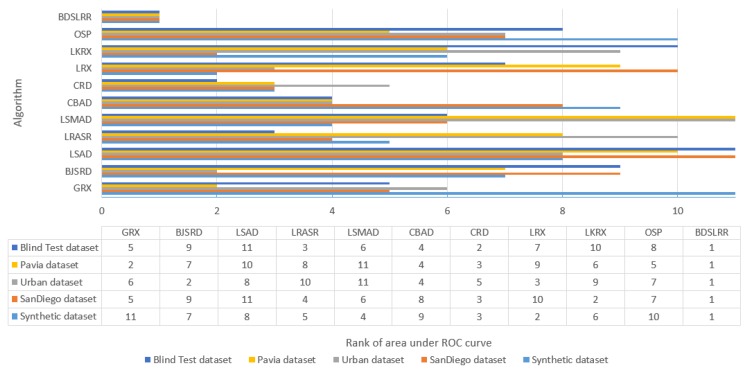
Rank of area under ROC curves for all datasets.

Moreover, from Table 1, Table 2, Table 3, Table 4 and Table 5, we can see that our SER and AER values are the lowest and highest, respectively, which proves that our proposed method achieves better measurement accuracy than all other algorithms in all datasets. Additionally, we compared the hyperspectral anomaly detection algorithms by comparing the rank of the area under ROC curves for all five datasets. Figure 24 shows that the proposed BDSLRR algorithm outperforms all other algorithms.

### 4.4. Parameter Analysis

This section examines the effect of parameters on detection performance of the proposed BDSLRR algorithm. The optimal parameters for all methods are shown in Table 6. As different methods will cause different detection results when choosing different parameters, to more fairly compare all detectors, we choose different parameters for different methods to obtain the best result.

The proposed method involves four parameters, namely the size of the patch, the number of clusters, the number of principal components, and λ. However, only the number of clusters needs to be tuned for each data, while the other parameters can be fixed to specific values in all experiments. In the following, we provide the setting details of those parameters.

#### 4.4.1. Patch Size

**Figure 25:**
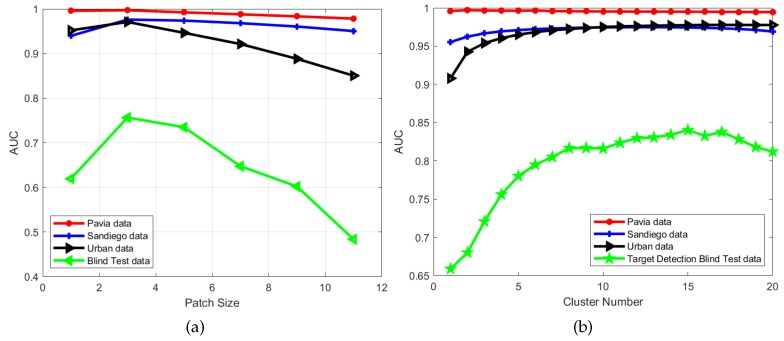
Parameter analysis for the patch size and cluster number for the proposed algorithm with the four real-world hyperspectral datasets. (**a**) AUC versus the patch size for the proposed algorithm; (**b**) AUC versus the cluster number for the proposed algorithm.

We first varied the patch size from 1×1 to 11×11 for the dictionary learning step, and the cluster number was set according to Table 6. As shown in Figure 25a, when the size is 1 (which means that there is no spatial–spectral constraint), detection performance is not optimal. However, when the size is too large, the detection performance can be decreased. Moreover, if the size is too large, it may entail a high computational burden. With experiments on different datasets, we can find that the best performance is obtained when the patch size is around 3. Thus, we fix the patch size as 3 in all experiments for simplicity.

#### 4.4.2. Cluster Number

For the cluster number of the background, we set the range from 1 to 20 for all real-world datasets. As shown in Figure 25b, when the number equals 1 (which means that the whole data is employed to learning the dictionary without cluster), the AUC value is not high in any of the datasets. As the cluster number increases, the AUC values also increase. Since the background of the Pavia data is less complex, a cluster number of 6 can reach the best result. As the San Diego and Urban data have a more complex background, the best performance is obtained when the cluster number is between 12 and 17, but it decreases when the number continually increases. Through our experiments, we found that, when the number cluster is between 6 and 20, the detection performance is stable.

#### 4.4.3. The Number of Principal Components

PCA components are utilized to construct the background dictionary. Through several rounds of experiences, we empirically found that the proposed method obtains the best performance when the number of the principal is 50. Thus, we fix the number of principal components as 50 in all experiments.

#### 4.4.4. λ

λ balances between the representation error and low-rank regularization. In the experiment, we found that the proposed method performs stably when lambda ranges from 0.001 to 0.005. For simplicity, we fix lambda as 0.002 in all experiments.

According to the discussion above, although the proposed method contains four parameters, only the cluster number needs to be manually tuned. Moreover, its best value often occurs around 10. Therefore, the proposed method is applicable to real applications.

#### 4.4.5. Computational Complexity of the Proposed Method

To evaluate the computational complexity of the proposed method, the running times of all methods were compared. Times are given in Table 7. All experiments were carried out with MATLAB software with 64-b Intel Core i7-7700k CPU 4.2-GHz and 16GB RAM. It is clear that, for all algorithms, as data size increases, running time increases. Compared with other methods, the time consumed by the proposed method is moderate and acceptable.

#### 4.4.6. Effectiveness of the Patch-Based Dictionary

**Figure 26:**
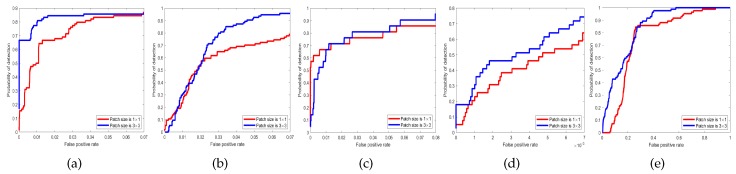
Without patch-based and patch-based ROC curves. (**a**) Synthetic Dataset; (**b**) San Diego Dataset; (**c**) Urban Dataset; (**d**) Pavia Dataset; (**e**) Target Detection Blind Test Dataset.

**Figure 27:**
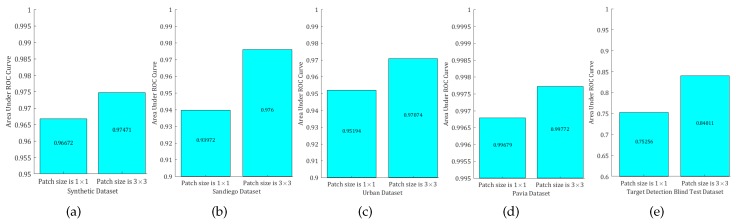
Without patch-based and patch-based AUC values. (**a**) Synthetic Dataset; (**b**) San Diego Dataset; (**c**) Urban Dataset; (**d**) Pavia Dataset; (**e**) Target Detection Blind Test Dataset.

To demonstrate the effectiveness of the patch-based dictionary, we performed experiments when patch size was 3×3 and 1×1 (which means that there is no spatial–spectral constraint) on the four datasets. Results are shown in Figure 26 and Figure 27. From the ROC curves and AUC values, we can see that our patch-based dictionary learning method can improve the detection performance since it acceptably explores the spatial–spectral characteristics.

## 5. Conclusions

This paper describes a new anomaly detection method for hyperspectral images. To more accurately represent the background, a patch-cluster-based cluster strategy is employed to exhibit the multi-mode structure of the background. The dictionary of each cluster was obtained utilizing the PCA method, and the whole background dictionary consisted of learning the sub-dictionary of each cluster. According to the cluster result, the multi-mode block-diagonal structure of the background is found when the data is represented by a learned dictionary. Based on the learned background dictionary, the block-diagonal structure-based low-rank representation model was built. After solving this model by employing the standard alternating direction method of multipliers (ADMM) algorithm, anomaly targets were extracted from the sparsity part. Since the proposed method integrates multi-mode block-diagonal structure information of the background for the HSI and local spatial–spectral feature into one-part, the detection performance is improved. Experiments on hyperspectral detection with four datasets and comparisons with other state-of-the-art detectors confirmed the superior performance of the proposed algorithm.

## Figures and Tables

**Table 1:** Synthetic Dataset: Assessment Metric Summary.

	GRX	BJSRD	LSAD	LRASR	LSMAD	CBAD	CRD	LRX	LKRX	OSP	BDSLRR
AUC	0.3965	0.8786	0.8603	0.9725	0.9745	0.8401	0.9747	0.9947	0.9548	0.3992	**0.9971**
Anomaly_error	74.9126	50.6298	31.9968	25.0568	42.7418	33.2518	38.3438	58.0259	45.2101	74.1839	**42.071**
Back_error	57.0853	1.0716	336.5827	90.7644	18.3331	552.9119	45.4601	0.0269	32.4945	62.3045	**4.7993**
SER	1.32	0.517	3.6858	1.1582	0.6107	5.8616	0.838	0.5805	0.777	1.3649	**0.4687**
ATPR	0.0566	0.2764	0.4266	0.5254	0.3452	0.5303	0.3951	0.205	0.3127	0.0612	**0.5892**
AFPR	0.0638	0.009	0.1658	0.0495	0.0099	0.1235	0.0248	0.0011	0.0207	0.068	**0.0044**
AER	0.9924	1.3696	1.4548	2.0025	1.5121	1.8659	1.6121	1.2566	1.4249	0.9928	**2.4236**

**Table 2:** San Diego Dataset: Assessment Metric Summary.

	GRX	BJSRD	LSAD	LRASR	LSMAD	CBAD	CRD	LRX	LKRX	OSP	BDSLRR
AUC	0.8082	0.7498	0.6456	0.8375	0.8082	0.7914	0.9404	0.7333	0.9569	0.796	**0.976**
Anomaly_error	83.8779	78.8697	16.3331	41.5007	69.2779	50.7018	80.1446	87.9746	54.019	82.8108	**40.4952**
Back_error	12.3604	4.602	1924.9814	213.8516	13.6146	371.7709	15.5111	8.1883	29.2151	15.2096	**48.119**
SER	1.5037	1.3042	30.333	3.9899	1.2952	6.6011	1.4946	1.5025	1.3005	1.5316	**1.2846**
ATPR	0.0555	0.0935	0.5913	0.3736	0.1682	0.3159	0.0769	0.0326	0.2698	0.0616	**0.353**
AFPR	0.0358	0.0138	0.5436	0.1503	0.0201	0.1674	0.0453	0.0265	0.0276	0.0402	**0.046**
AER	1.5037	1.0879	1.1165	1.3565	1.1781	1.217	1.0343	1.0063	1.3317	1.0228	**1.4745**

**Table 3:** Urban Dataset: Assessment Metric Summary.

	GRX	BJSRD	LSAD	LRASR	LSMAD	CBAD	CRD	LRX	LKRX	OSP	BDSLRR
AUC	0.9347	0.9544	0.9284	0.8963	0.8426	0.9432	0.9377	0.9309	0.9152	0.9338	**0.9707**
Anomaly_error	11.4441	12.9748	8.544	5.472	9.3439	5.8013	20.3721	5.5153	9.2795	11.5167	**12.6175**
Back_error	29.0146	1.844	218.1306	774.0153	204.3909	329.3714	2.8357	163.467	59.0881	28.9068	**0.4959**
SER	0.5057	0.1852	2.8334	9.7436	2.6717	4.1897	0.2901	2.1123	0.8546	0.5053	**0.1639**
ATPR	0.2782	0.2495	0.3852	0.5118	0.3836	0.5134	0.0152	0.5715	0.3754	0.2756	**0.5592**
AFPR	0.0452	0.0129	0.1393	0.2673	0.1014	0.1388	0.0022	0.0976	0.0449	0.0451	**0.0024**
AER	1.3229	1.3153	1.3998	1.5007	1.4579	1.7699	1.0131	2.1058	1.5291	1.3183	**2.2632**

**Table 4:** Pavia Dataset: Assessment Metric Summary.

	GRX	BJSRD	LSAD	LRASR	LSMAD	CBAD	CRD	LRX	LKRX	OSP	BDSLRR
AUC	0.996	0.9917	0.9701	0.9894	0.9678	0.9956	0.996	0.9757	0.9934	0.995	**0.9977**
Anomaly_error	12.1911	32.0714	23.1599	22.2364	27.8242	2.7859	20.1087	29.4959	17.0997	27.4503	**26.5708**
Back_error	30.7702	1.4285	82.742	53.8645	2.5432	225.5181	74.9304	6.5857	5.269	11.806	**8.7455**
SER	0.3113	0.2428	0.7674	0.5515	0.2201	1.6544	0.6887	0.2615	0.1621	0.2845	**0.2059**
ATPR	0.4852	0.094	0.2392	0.265	0.1751	0.168	0.307	0.1551	0.3916	0.1729	**0.4989**
AFPR	0.0214	0.0093	0.0761	0.0594	0.0059	0.0586	0.069	0.0148	0.0043	0.0272	**0.0229**
AER	1.9008	1.0934	1.2144	1.2796	1.2052	1.1315	1.3435	1.1661	1.6366	1.1762	**1.9499**

**Table 5:** Blind Test Dataset: Assessment Metric Summary.

	GRX	BJSRD	LSAD	LRASR	LSMAD	CBAD	CRD	LRX	LKRX	OSP	BDSLRR
AUC	0.6833	0.5124	0.2825	0.7611	0.7404	0.7564	0.7811	0.7281	0.4133	0.7044	**0.8401**
Anomaly_error	82.9128	82.3548	48.3462	31.1976	80.6864	78.4242	78.0669	80.0454	83.3869	80.5666	**79.7723**
Back_error	77.9783	37.0136	6343.4578	6634.3314	101.1229	123.2394	38.425	8.4918	112.602	35.9273	**35.9873**
SER	0.1738	0.1289	6.9027	7.1984	0.1963	0.2178	0.1258	0.1956	0.2117	0.1258	**0.1150**
ATPR	0.0066	0.0099	0.2423	0.406	0.02	0.0344	0.0367	0.0287	0.0036	0.0209	**0.3258**
AFPR	0.0039	0.0125	0.2604	0.219	0.0149	0.0164	0.0141	0.005	0.013	0.0101	**0.0101**
AER	1.0026	0.9974	0.976	1.3147	1.0052	1.0187	1.0234	1.0244	0.9906	1.0111	**1.4683**

**Table 6:** Optimal Parameters of All Methods for All Datasets.

	LSMAD		LRASR		BJSRD		LSAD	CBAD	CRD		LRX		LKRX		BDSLRR	
	Rank	Card	Cluster Number	P	Inner Window	Outer Window	Neighbors’ Number	Cluster Number	Inner Window	Outer Window	Inner Window	Outer Window	Inner Window	Outer Window	Patch Size	Cluster Number
Synthetic dataset	5	0.044	15	75	7	19	31	10	9	11	5	7	5	7	3	12
San Diego dataset	3	0.075	19	60	15	19	7	10	7	13	7	9	7	9	3	12
Urban dataset	3	0.075	5	80	3	5	19	9	7	15	7	11	7	11	3	17
Pavia dataset	3	0.075	6	80	3	5	9	8	7	9	3	5	3	5	3	6
Blind Test dataset	16	0.001	13	10	15	17	7	13	7	9	5	7	5	7	3	20

**Table 7:** Running Times (s) of All Methods for All Datasets.

	GRX	BJSRD	LSAD	LRASR	LSMAD	CBAD	CRD	LRX	LKRX	OSP	BDSLRR
Synthetic dataset	0.26	854.42	410.16	4.48	15.37	0.86	74.85	13.57	18.57	0.40	**68.79**
San Diego dataset	0.15	449.02	114.06	2.99	9.87	0.59	68.5	6.72	11.43	0.80	**44.77**
Urban dataset	0.18	359.75	153.17	3.08	12.58	0.66	114.19	7.65	11.45	0.93	**60.41**
Pavia dataset	0.25	434.31	105.51	7.47	14.04	0.91	35.15	16.25	17.11	0.99	**45.03**
Blind Test dataset	39.01	2269.17	894.34	9.42	139.51	5.77	294.89	65.41	77.61	0.82	**191.72**
